# 

**Published:** 1895-01-19

**Authors:** 


**Notices of Births, Deaths, and Marriages** are inserted in this column at a charge of 2s. 6d. for an announcement not exceeding four lines.

**Notice to Advertisers and Subscribers.**

All Advertisements, Orders for Copies of THE HOSPITAL, and Business Communications should be addressed to **The Manager, NOT TO THE EDITOR,** THE HOSPITAL, 428, Strand, London, W.C.

The Cost of Subscription, post free, is as follows:—Per week, 2½d.; per quarter, 2s. 8d.; per half-year, 5s. 3d.; per annum, 10s. 6d. Foreign:—Per Annum, 15s. 2d.; payable, in all cases, in advance.

### NOTICE TO CORRESPONDENTS.

All MS., Letters, Books for Review, and other matters intended for the Editor should be addressed THE EDITOR, THE LODGE, PORCHESTER SQUARE, LONDON, W.

The Editor cannot undertake to return rejected MS., even when accompanied by stamped directed envelope.

**"Burdett's Hospital Annual," 1895,**

IN ACTIVE PREPARATION.

Sixth year of publication. About 900 pp. Scarlet cloth, gilt lettered. Price 5s. A most complete guide to British, Colonial, Indian, and American Hospitals, Dispensaries, Nursing and Convalescent Institutions, and Asylums. A few copies of the "Annual" for 1894 may still be obtained on application to the Publishers, THE SCIENTIFIC PRESS (Limited), 428, Strand, London, W.C.

**NOTICE.—The Index for Vol. XVI. (ending September 24th, 1894) is on sale at the Office of this Journal, Price 2½d., post free.**

### THE STUDENT OF MEDICINE.

### APPOINTMENTS.

H. E. Brodrick, M.D., B.S., M.R.C.S., L.S.A.Lond., appointed Honorary Medical Officer to the Richmond (Surrey) Dispensary. D. W. Inglis, M.D., appointed Medical Officer of the Hebburn District of the South Shields Union. H. R. Kenwood, M.B., C.M.Edin., L.R.C.P.Lond., reappointed Medical Officer of Health to the Finchley Local Board. T. B. Law, M.B., C.M., appointed a District Medical Officer in the Colonial Medical Service at Nicosia, Cyprus. O. E. B. Marsh, L.R.C.P.Edin., M.R.C.S.Eng., appointed Honorary Surgeon to the In-patients at the Newport Infirmary. H. J. Palmer, L.R.C.P., L.R.C.S. Edin., L.M., L.F.P.S.Glasg., appointed Medical Officer of the Heath, Sutton-cum-Duckmanton, and Temple Normanton District. Amy Sheppard, M.B.Lond., appointed Assistant Physician to the New Hospital for Women. A. P. Steavenson, M.B., C.M.Edin., appointed Senior House Surgeon to the District Hospital, West Bromwich. W. F. Umnev, M.D.Lond., M.R.C.S. Eng., appointed Assistant Medical Officer to the Home and Infirmary for Sick Children, Sydenham, S.E. F. J. Wadham, L.R.C.P.Lond., M.R.C.S.Eng., appointed a Medical Officer to the Royal Isle of Wight Infirmary, Ryde. Miss Mabel Jones, M.B., appointed Assistant Anæsthetist to the Royal Free Hospital, Gray's Inn Road. Miss L. B. Aldrich-Blake, M.D., B.S.Lond., appointed Anæsthetist to the Royal Free Hospital, Gray's Inn Road.

### VACANCIES.

The following vacancies are announced this week, the amount of salary in each case being given after the name of the institution:

#### Resident Medical Officers.

City of Dublin Hospital.—House Surgeon. Salary £50 a year, with apartments, light, fuel, and attendance. Applications to the Honorary Secretary, Medical Board, City of Dublin Hospital, Upper Baggot Street, Dublin, by January 19th.

Guest Hospital, Dudley.—Resident Medical Officer; doubly qualified. Salary commencing £100 per annum, increasing £10 a year to £120 if services are satisfactory, with board, residence, attendance, and washing. Applications to the Secretary by January 24th.

Hospital for Consumption and Diseases of the Chest, Brompton.—Resident House Physicians. Applications to the Secretary by January 24th.

Royal Hospital, Chelsea.—Dispenser; must be on the *Medical Register*, preference given to a married man with qualifications in surgery. Remuneration 10s. a day, with unfurnished quarters, fuel, and light. Applications to the Secretary by January 19th.

#### Non-Resident Medical Officers.

Newton Abbot Union.—Medical Officer and Public Vaccinator for the Ashburton (No. 1) District. Salary £72 10s. per annum and £3 per annum for the medical officer to provide a surgery at Broadhempston. Salary mentioned will include all extras except payment of 10s. 6d. for each midwifery attended and the usual payment for vaccination. Applications to John Alsop, Clerk, Union Offices, Newton Abbot. Election on January 23rd.

University of Edinburgh.—Additional Examiner in Natural History and Clinical Surgery. Period of office four years. Salary £75 per annum in each case, with £10 per annum for travelling and other expenses in the case of an additional examiner not resident in Edinburgh or the immediate neighbourhood. An additional allowance is made to the Examiner in Natural History for examining for Graduates in Arts. Applications to L. J. Grant, Interim Secretary, University Court, University of Edinburgh, by February 6th.

### UNIVERSITIES AND COLLEGES.

EXAMINING BOARD IN ENGLAND BY THE ROYAL COLLEGES OF PHYSICIANS AND SURGEONS.

THE following gentlemen passed the Second Examination of the Board in the subjects indicated at a meeting of the Examiners, on Monday, January 7th.

PASSED IN PHYSIOLOGY ONLY.—A. Cubley, of Firth College, Sheffield, and Mr. Cooke's School of Anatomy and Physiology; F. C. Torbitt, of Owens College, Manchester; A. Evans, of St. Mungo's College, Glasgow: G. H. Heron, of University College, Liverpool; J. Gott, of King's College, London; J. B. A. Trensch, of Guy's Hospital; and M. C. B. Anderson, of St. Mary's Hospital.

THE STUDENT OF MEDICINE—*continued.*

Passed in Anatomy and Physiology.—H. O. P. De Mirimonde, student of Yorkshire College, Leeds; N. J. Kendal, W. A. L. Jackson, and J. C. Young, of Mason College, Birmingham; F. T. Butler, R. Hughes, H. M. Rees, W. H. Stott, and F. S. Rhodes, of Owens College, Manchester; E. Symes and G. W. B. Beverley, of University College, Bristol; D. R. Edwards, C. J. L. Palmer, and W. Daley, of University College, Liverpool; R. R. Horley, of Middlesex Hospital; and J. Templeton, of St. Mary's Hospital.

Passed in Anatomy only.—L. N. Lloyd, of Charing Cross Hospital; H. R. Rice, of Mason College, Birmingham, and London Hospital; C. D. Ingle, of University College, Bristol; T. P. Yates, of Owens College, Manchester; and S. H. Longhurst, of Guy's Hospital.

Thirteen gentlemen were referred in both subjects, and five in Anatomy only.

Tuesday, January 8th:

Passed in Anatomy and Physiology.—W. Q. Bown, W. R. Flint, and C. H. Furnival, students of St. Mary's Hospital; E. H. Ross, E. L. Forward, W. S. Maugham, and H. S. E. Williams, of St. Thomas's Hospital; R. G. Murray, of St. George's Hospital; J. Oldfield, G. A. W. Spear, and H. J. Hutchens, of St. Bartholomew's Hospital; T. A. Dowse, of Charing Cross Hospital; G. Q. Richardson, of Adelaide Hospital and Royal University, Dublin; A. E. Gilmour and S. E. Denyer, of Cambridge University; F. J. Nicholls and R. Balderston, of Guy's Hospital.

Passed in Anatomy Only.—D. Ackland and J. H. C. Fegan, of Charing Cross Hospital; F. Voller, of St. Thomas's Hospital; T. H. Bailey, of King's College, London, and Mr. Cooke's School of Anatomy and Physiology; and R. D. Dobie, of King's College, London.

Passed in Physiology Only.—S. H. Mason and C. C. Poole, of Guy's Hospital; G. F. M. Clarke, of Charing Cross Hospital; C. N. Chadborn, of St. George's Hospital; and E. F. Crabtree, of St. Bartholomew's Hospital.

Eleven gentlemen were referred in both subjects, five in Anatomy only, and five in Physiology only.

Wednesday, January 9th:

Passed in Anatomy and Physiology.—H. A. C. Harris and A. C. Haslam, students of St. Thomas's Hospital; G. O'Neill, of Charing Cross Hospital; B. F. Wingate, of St. Mary's Hospital; J. B. Walters, J. G. Watt, and C. C. Worts, of Guy's Hospital; F. Buckwell, W. Beckton, and E. G. Klumpp, of St. Bartholomew's Hospital: A. S. Bruzand and H. J. Bryan, of London Hospital; and H. Breton, of St. George's Hospital.

Passed in Anatomy only.—A. R. O'Ffiahertie, of Queen's College, Cork, and London Hospital; C. A. C. Salmon, of Guy's Hospital; F. A. Pitts Tucker, of St. Thomas's Hospital; and C. S. Agnew, of Charing Cross Hospital and Mr. Cooke's School of Anatomy and Physiology.

Passed in Physiology only.—R. L. Argles, of St. Mary's Hospital and Mr. Cooke's School of Anatomy and Physiology; A. J. Andrew, of St. Bartholomew's Hospital; and A. E. Malaher, of St. Thomas's Hospital.

Sixteen gentlemen were referred in both subjects, six in Anatomy only, and four in Physiology only.

SOCIETY OF APOTHECARIES OF LONDON.

Primary Examination, Part II., January, 1895.

The following candidates passed:—

Anatomy and Physiology.—C. S. Agnew, Charing Cross; W. Amsden, St. Bartholomew's; E. Brice, Birmingham; E. F. Crabtree, St. Bartholomew's; E. F. Dowding, St. Thomas's; W. H. Gale, St. Thomas's; A. H. Gibbon, St. Thomas's; A. T. Griffiths, Birmingham; H. W. Hues, Birmingham; T. T. Hughes, Middlesex; W. J. Lindsay, Guy's; M. Paine, Women's Medical College; C C. Poole, Guy's; E. W. W. Pughe, Birmingham; C. B. Salway, St. Thomas's; H. E. Scowcroft, Cambridge; E. Whalley, Leeds; A. M. St. J. Wright, Madras; L. Wright, St. Thomas's.

Anatomy.—T. H. Bailey, King's; P. Cator, St. Bartholomew's; C. F. W. Dunn, Cambridge; A. H. Fitzgibbon, St. Bartholomew's; C. F. Eddowes, St. George's; J. G. Gowlland, St. George's; M. B. Hebron, King's; H. M. Maitland, Women's Medical College; S. J. Meredith, Birmingham; A. Ross, Guy's; J. Scarr, Manchester; A. M. Williams, Women's Medical College.

Physiology.—G. F. M. Clarke, Charing Cross; J. Freeman, University College, Bristol; J. R. Jeaffreson, St. Bartholomew's; F. T. Knott, Guy's; H. M. Waller, St. Bartholomew's.

Primary Examination, Part I.

Chemistry, Materia Medica, Botany, and Pharmacy.—H. M. Hardy, South London School of Pharmacy.

Chemistry.—L. Bradstock, Birmingham; C. H. F. Dalton, Charing Cross; A. G. Lang, Guy's.

Materia Medica and Pharmacy.—C. F. Eddowes, St. George's; A. C. McLean, King's.

Materia Medica.—W. O. Piper, Westminster.

Biology.—S. K. K. Haslam and E. C. Scarlett, Women's Medical College.

The diploma of this Society entitles the holder to practice Medicine, Surgery, and Midwifery.

